# AZD6738 Inhibits fibrotic response of conjunctival fibroblasts by regulating checkpoint kinase 1/*P53* and *PI3K*/*AKT* pathways

**DOI:** 10.3389/fphar.2022.990401

**Published:** 2022-09-20

**Authors:** Longxiang Huang, Qin Ye, Chunlin Lan, Xiaohui Wang, Yihua Zhu

**Affiliations:** ^1^ The First Affiliated Hospital of Fujian Medical University, Fuzhou, China; ^2^ Fujian Medical University Union Hospital, Fuzhou, China

**Keywords:** AZD6738 (ceralasertib), ATR inhibitor, human conjunctival fibroblasts (HConFs), subconjunctival fibrosis, apoptosis

## Abstract

Trabeculectomy can effectively reduce intraocular pressure (IOP) in glaucoma patients, the long-term surgical failure is due to the excessive proliferation and fibrotic response of conjunctival fibroblasts which causes the subconjunctival scar and non-functional filtering bleb. In this study, we demonstrated that AZD6738 (Ceralasertib), a novel potent ataxia telangiectasia and Rad3-related (ATR) kinase inhibitor, can inhibit the fibrotic response of conjunctival fibroblasts for the first time. Our *in vitro* study demonstrated that AZD6738 inhibited the level and the phosphorylation of checkpoint kinase 1 (CHK1), reduced TGF-β1-induced cell proliferation and migration, and induced apoptosis of human conjunctival fibroblasts (HConFs) in the high-dose group (5 μM). Low-dose AZD6738 (0.1 μM) inhibited the phosphorylation of CHK1 and reduce fibrotic response but did not promote apoptosis of HConFs. Further molecular research indicated that AZD6738 regulates survival and apoptosis of HConFs by balancing the CHK1/P53 and PI3K/AKT pathways, and inhibiting TGF-β1-induced fibrotic response including myofibroblast activation and relative extracellular matrix (ECM) protein synthesis such as fibronectin (FN), collagen Ⅰ (COL1) and collagen Ⅳ (COL4) through a dual pharmacological mechanism. Hence, our results show that AZD6738 inhibits fibrotic responses in cultured HConFs *in vitro* and may become a potential therapeutic option for anti-subconjunctival scarring after trabeculectomy.

## Introduction

Glaucoma, characterized by elevated intraocular pressure and progressive dysfunction and death of retinal ganglion cells and their axons, is the second leading cause of blindness after cataracts (Davis et al., 2016). At present, lowering intraocular pressure (IOP) remains the primary clinical treatment although IOP is not the only factor contributing to glaucoma (Quigley and Broman, 2006). Laser or surgery is still required with the effective interventions for glaucoma when drug therapy does not achieve sufficient IOP reduction and manageable retinal ganglion cell damage (Weinreb et al., 2014; Lusthaus and Goldberg, 2019). Although minimally invasive glaucoma surgeries (MIGS) or gene therapy have shown to be effective interventions for glaucoma (King et al., 2018; Tan et al., 2019; Mathew and Buys, 2020; Tan et al., 2020), glaucoma filtration surgeries remain the first-choice and standard surgical treatment for many types of glaucoma, which are widely implemented in clinical (Cairns, 1968; Eldaly et al., 2014). Trabeculectomy, a type of glaucoma filtration surgery, is the most commonly used surgical procedure for reducing IOP in patients with glaucoma (Gedde et al., 2018).

The success of trabeculectomy mainly depends on the functional filtering bleb formed during the postoperative wound-healing process (Addicks et al., 1983), but the long-term successful rate of this operation is not always satisfactory due to the excessive proliferation of fibroblasts or subconjunctival fibrosis during the healing period that causes the tissue to scar and leads subsequently to the function loss of the filtering bleb (Addicks et al., 1983; Schlunck et al., 2016; Zada et al., 2018). The underlying mechanism of subconjunctival fibrosis is currently not fully elucidated. Conjunctival fibroblasts differentiate into myofibroblasts after surgery, migrate and proliferate, and synthesize extracellular matrix (ECM) proteins including several types of collagen (COL) fibers, laminins (LN), and fibronectin (FN), which lead to the formation of scar tissue at the surgical site (Shu and Lovicu, 2017; NikhalaShree et al., 2019). At present, antiproliferative agents such as mitomycin C (MMC) and 5-fluorouracil (5-FU) have been used to counteract the proliferative activity of cells to prevent subconjunctival fibrosis after trabeculectomy (Kitazawa et al., 1991; Green et al., 2014; Cabourne et al., 2015; Bell et al., 2021). However, such drugs can cause serious complications related to non-specific cytotoxicities (e.g. blepharitis, keratitis, bleb leakage, endophthalmitis, or bleb-related infections) (Franks and Hitchings, 1991; Mearza and Aslanides, 2007). Therefore, a more targeted and safer anti-fibrotic drug that can be used after glaucoma filtration surgery is critical to ensure the preservation of bleb function and a healthy ocular state postoperation.

AZD6738 (Ceralasertib), a novel potent ataxia telangiectasia and Rad3-related (ATR) kinase inhibitor, can lead to cell death and senescence in several cancer cells including non-small cell lung cancer cell lines and human breast cancer cell lines, and down-regulates DNA damage-and cell proliferative-related genes (Vendetti et al., 2015; Kim et al., 2017). ATR and ATM belong to a family of kinases that respond to DNA strand breaks caused by destructive or normal processes. ATR or ATM can phosphorylate and activate a variety of substrates after being activated, including checkpoint kinase 1 (CHK1), checkpoint kinase 2 (CHK2), tumor suppressors such as P53, DNA repair factors such as Rad50, GADD45. CHK1, a major substrate of ATR, is required for checkpoint-mediated cell cycle arrest and activation of DNA repair in response to the presence of DNA damage or unreplicated DNA. Cell cycle arrest in the G1 phase is mediated by P53, which is a substrate of CHK1 and ATR (Feijoo et al., 2001; Heffernan et al., 2002). Recent research showed that CHK1 is a key regulator of lung fibrosis and CHK1 inhibition is considered as a potential novel therapeutic option for idiopathic pulmonary fibrosis (Wu et al., 2022). Based on the clue, we hypothesized that CHK1 inhibitors have a potential role in subconjunctival fibrosis after trabeculectomy.

Therefore, this study was to determine the effects of AZD6738 on cultured primary human conjunctival fibroblasts (HConFs) *in vitro* and whether it could counteract TGF-β1-induced fibrosis in HConFs. To accomplish this, we examined the morphological and biological activities of HConFs induced by different concentrations of AZD6738 and TGF-β1.

## Materials and methods

### Cell culture and treatment paradigms

Primary HConFs (Cat NO. 6570; ScienCell Research Laboratories, Inc., Carlsbad, CA, United States) were isolated from human conjunctiva and grown at 37°C in a humidified atmosphere of 95% air and 5% CO_2_ incubator and were maintained in DMEM/F12 1:1 medium (PM150314; Procell Life Science and Technology Co., Ltd., Wuhan, China) with 10% FBS which was replaced once on alternate days. The cultured HConFs passed the short tandem repeat (STR) identification carried out by Sixin Biotechnology Co., Ltd. (Shanghai, China). The specific detection protocol is as follows: DNA was extracted by using an Axygen genome extraction kit, amplified by 21-STR amplification protocol, and the STR loci and the Amelogenin sex gene were detected on an ABI 3730XL STR genetic analyzer. Cells in passages 3 to 6 were used in all experiments. The cells were treated with 0.1, 0.25, 0.5, 1, 2, 5 and 10 μM AZD6738 (Cat No. HY-19323; MedChemExpress, NJ, United States), 0.1% dimethyl sulfoxide (DMSO; diluted with cell culture medium) and 10 ng/ml TGF-β1 (PeproTech, NJ, United States) for 24 or 48 h. For the group treated with 0.1 and 5 μM AZD6738, cells were treated with 10 ng/ml TGF-β1 (Willard et al., 2020; Correia-Sá et al., 2021), treatment for 48 h simultaneously. The blank control group was set in all experiments, cells were treated with a culture medium only. AZD6738 was dissolved in DMSO, while TGF-β1 was dissolved in distilled water. All the drugs were diluted in the cell culture medium to achieve their working concentrations. The morphology images of HConFs were taken under a Leica DM4 microscope (DM400B; Leica, Wetzlar, Germany). The same number of HConFs seeded in 96-well plates were counted under a 100 × inverted microscope every 12 h after being treated with 1 and 5 μM AZD6738. Six replicate wells were repeated for each group and the average value was calculated for drawing the cell growth curve.

### Cell viability analysis

Cell Counting Kit-8 (CCK-8) assay kit (Dojindo Molecular Technologies, Kumamoto, Japan) and CytoTox 96 Non-Radioactive Cytotoxicity Assay (Promega, Fitchburg, WI) were used to detect the viability and cell metabolic ability. HConFs were seeded in 96-well plates at 5×10^3^ cells per well and treated with 0.25, 0.5, 1, 2, 5 and 10 µM AZD6738 or 10 ng/ml TGF-β1 for 24 or 48 h respectively. Six replicate wells were repeated for each group. 50μL original culture medium was transferred to a new 96-well plate for LDH release test according to the manufacturer’s instructions, the absorbance was measured at 492 nm using a flame atomic absorption spectrophotometry (PerkinElmer, Boston, MA, United States). The surplus original culture medium was discarded and a new culture medium containing 10 µL CCK-8 reagent was added to each well. After incubation for 3 h, the absorbance was measured at 450 nm.

### Migration assay

HConFs were seeded in 24-well plates at 2×10^4^ cells per well for 24 h to reach about 80%–90% confluence. The cells were scratched with the tip of the sterilized 1 ml pipette and washed the debris after being treated with 5 μM AZD6738 and 10 ng/ml TGF-β1. Images were taken at 0 h, 24 h, and 48 h after scratching respectively, and analyzed by ImageJ (National Institutes of Health, Maryland, United States). The percentage of wound healing was calculated with the following formula: (initial wound area - unhealed wound Area)/initial wound area × 100% (Santos et al., 2015).

### Assay of collagen gel contraction

HConFs were harvested by exposure to trypsin-EDTA, washed twice, and resuspended in serum-free DMEM/F12 medium. Type I collagen from rat tail tendon (C8062, 5 mg/ml; Solarbio Science and Technology Co., Ltd., Beijing, China), 10× PBS, 0.06 × 0.1 mol/L NaOH, and HConFs suspension were mixed on ice in an appropriate volume ratio (final concentration of type I collagen, 1 mg/ml; final cell density, 2.5 × 10^5^ cells/ml). A portion (0.5 ml) of the mixture was added to each well of the 24-well culture plates and allowed to solidify by incubation at room temperature for 20 min. The collagen gels were freed from the sides of the wells with a sterilized 10-μL pipette. Serum-free medium (0.5 ml) containing 10 ng/ml TGF-β1 or 5 µM AZD6738 was added to the surface of each corresponding gel. Represent images of collagen gel contraction were photographed by a Fluor ChemE (92–14860–00; ProteinSimple, San Jose, CA, United States). The gel size was measured by ImageJ. The percentage of contraction area of each group was calculated as follows: (initial gel area - gel area at each time point)/initial gel area × 100% (Lan et al., 2021).

### Measurement of reactive oxygen species

Reactive oxygen species (ROS) was detected using a OxiSelect™ *In Vitro* ROS/RNS Assay Kit (LOT: 7081363, Cell Biolabs, Inc., San Diego, CA, United States). HConFs were cultured and treated in 24-well plates, and then stained with DCFH-DA at 37 °C for 30 min. After three washes with PBS, the intensity of the fluorescence was measured using a fluorescence microscope (DM400B; Leica, Wetzlar, Germany), the result were analyzed by ImageJ and quantified by relative fluorescence intensity.

### Immunofluorescence and TUNEL analysis

Cells climbing slices were fixed with 4% PFA at room temperature (RT) for 10 min and further incubated with 5% BSA and 0.5% Triton X-100 at RT for 1 h. For immunofluorescence, cells were incubated at 4°C overnight with the primary antibodies against FN (TA502601, mouse monoclonal antibody, 1:200; OriGene Technologies Inc., Rockville, MD, United States), α-SMA (ab7817, mouse monoclonal antibody, 1:100; Abcam, Cambridge, MA, United States), COL1A1 (ab6308, mouse monoclonal antibody, 1:250; Abcam, Cambridge, MA, United States), COL4A3 (ab111742, rabbit polyclonal antibody, 1:250; Abcam, Cambridge, MA, United States) and Cleaved-Caspase3 (9,661, rabbit monoclonal antibody, 1:200; Cell Signaling Technology, Danvers, MA, United States) followed by incubation with Alexa Fluor 488 goat anti-mouse or Alexa Fluor 488 goat anti-rabbit (1:1,000; Invitrogen, Carlsbad, CA, United States). For F-actin staining, cells were incubated with Rhodamine-phalloidin (PHDR1, 1:1,000; Cytoskeleton, Denver, CO, United States) at room temperature for 30 min. Subsequently, the cell climbing slices were taken out and counterstained with DAPI (F6057-20ML, Sigma, MO, United States) for 5 min, and visualized under a fluorescence microscope. The results were analyzed by ImageJ and quantified by relative fluorescence intensity.

For terminal deoxynucleotidyl transferase-mediated Nick end labeling (TUNEL) staining, after being treated with 1 and 5 μM AZD6738 and 10 ng/ml TGF-β1, cells were processed with a TUNEL kit (11684817910; Roche, Switzerland) according to the product manual and further counterstained with DAPI for 5 min.

### Western blot

HConFs seeded in two 12-well plates were collected after being treated with 0.1 and 5 μM AZD6738 and 10 ng/ml TGF-β1 for 48 h. Total proteins were extracted from HConFs using radio immunoprecipitation assay (RIPA) lysis buffer (CWBio, Beijing, China) containing 1% protease inhibitor cocktail (Sigma-Aldrich). After incubation on ice for 30 min, debris was removed by centrifugation (12,000 g) at 4 C and protein concentration was quantified by BCA assay. Proteins were diluted in 5× loading buffer and denatured at 98 C for 3 min 20 μg protein samples were fractionated by SDS-PAGE using the commercial polyacrylamide gel (Bio-Rad, Hercules, CA, United States) and transferred onto polyvinylidene fluoride membrane (Millipore, Burlington, MA, United States). Membranes were blocked in 5% nonfat dried milk in 1× Tris-buffered saline with 0.1% Tween-20 (TBS-T) at room temperature for 1 h. Subsequently, membranes were incubated with the indicated primary antibodies against CHK1 (ab32531, rabbit monoclonal antibody, 1:1,000; Abcam, Cambridge, MA, United States), p-CHK1 (ab283261, phospho S345, rabbit monoclonal antibody, 1:1,000; Abcam, Cambridge, MA, United States), MMP9 (ab76003, mouse monoclonal antibody, 1:1,000; Abcam, Cambridge, MA, United States), SMAD3 (#9523, Rabbit monoclonal antibody, 1:1,000; Cell Signaling Technology, Danvers, MA, United States), p-SMAD2/3 (#8828, Rabbit monoclonal antibody, 1:1,000; Cell Signaling Technology, Danvers, MA, United States), p-eNOS (9,574, phospho T495, rabbit monoclonal antibody, 1:1,000; Cell Signaling Technology, Danvers, MA, United States), RhoA (2,117, rabbit monoclonal antibody, 1:1,000; Cell Signaling Technology, Danvers, MA, United States), FN (ab6328, mouse monoclonal antibody, 1:1,000; Abcam, cambridge, MA, United States), CDC2 (9116S, mouse monoclonal antibody, 1:1,000; Cell Signaling Technology, Danvers, MA, United States), C-MYC (ab32072, rabbit monoclonal antibody, 1:1,000; Abcam, cambridge, MA, United States), α-SMA (ab7817, mouse monoclonal antibody, 1:1,000; Abcam, Cambridge, MA, United States), β-Catenin (ab32572, rabbit monoclonal antibody, 1:1,000; Abcam, cambridge, MA, United States), GAPDH (AF5718, goat polyclonal antibody, 1 mg/ml; R&D Systems, Minneapolis, MN, United States), and α-Tubulin (ab176560, rabbit monoclonal antibody, 1:1,000; Abcam, Cambridge, MA, United States) overnight at 4 °C. After washing with 1 × TBS-T for 30 min, membranes were incubated with corresponding secondary antibodies (1:10,000; ZSGB-Bio) at room temperature for 1 h. Bands were visualized with a Fluor ChemE (ProteinSimple) and ImageJ was used to analyze the gray value of each protein band. All experiments were done were done in a single time with at least three replicates.

### Quantitative PCR

HConFs seeded in two 24-well plates were collected after being treated with 0.1 and 5 μM AZD6738 and 10 ng/ml TGF-β1 for 48 h. Total RNA was extracted from cultured HConFs with Trizol Reagent (Invitrogen, CA, United States). The absorbance values of RNA at OD 260 and OD 280 were detected using a spectrophotometer (Biophotometer plus, Eppendorf, Germany) to calculate the RNA content, and reverse transcription reaction was conducted using a kit (RevertAid First Strand cDNA Synthesis Kit; Fermentas, Thermo Fisher Scientific, Pittsburgh, PA, United States). SYBR Green Real-Time PCR Master Mix (Toyobo, Osaka, Japan) was used for quantitative real-time PCR on a Rotor-Gene Q cycler (QIAGEN, Germantown, MD, United States). Each reaction was run in triplicate. Transcript abundance was reported as relative to Gapdh levels and was calculated using the 2^−ΔΔCt^ method. qPCR was performed in three technical replicates on each biological replicate. All experiments were done were done in a single time with at least three replicates. The primer pairs used in this study are listed as follows: *CHK1* sense, ATA​TGA​AGC​GTG​CCG​TAG​ACT; antisense, TGC​CTA​TGT​CTG​GCT​CTA​TTC​TG; *FN* sense, CGG​TGG​CTG​TCA​GTC​AAA​G; antisense, AAA​CCT​CGG​CTT​CCT​CCA​TAA; *α-SMA* sense, GGC​ATT​CAC​GAG​ACC​ACC​TAC; antisense, CGA​CAT​GAC​GTT​GTT​GGC​ATA​C; *VEGFA* sense, AGG​GCA​GAA​TCA​TCA​CGA​AGT; antisense, AGG​GTC​TCG​ATT​GGA​TGG​CA; *MMP9* sense, TGT​ACC​GCT​ATG​GTT​ACA​CTC​G; antisense, GGC​AGG​GAC​AGT​TGC​TTC​T; *P53* sense, CAG​CAC​ATG​ACG​GAG​GTT​GT; antisense, TCA​TCC​AAA​TAC​TCC​ACA​CGC; *AKT* sense, AGC​GAC​GTG​GCT​ATT​GTG​AAG​T; antisense, GCC​ATC​ATT​CTT​GAG​GAG​GAA​GT; *Caspase3* sense, CAT​GGA​AGC​GAA​TCA​ATG​GAC​T; antisense, CTG​TAC​CAG​ACC​GAG​ATG​TCA; *BAX* sense, CCC​GAG​AGG​TCT​TTT​TCC​GAG; antisense, CCA​GCC​CAT​GAT​GGT​TCT​GAT; *BCL2* sense, GGT​GGG​GTC​ATG​TGT​GTG​G; antisense, CGG​TTC​AGG​TAC​TCA​GTC​ATC​C; *GAPDH* sense, ACA​GTC​AGC​CGC​ATC​TTC​TT; antisense, ACG​ACC​AAA​TCC​GTT​GAC​TC.

### Statistics

All data were analyzed using SPSS 26 software (IBM-SPSS 26. 0. 0. 0; Chicago, IL, United States). Experiments involving 2 groups were compared using an unpaired, 2-tailed *t*-test. One-way ANOVA was used for comparison among three or more groups, followed by an LSD post hoc test for homogeneous variances or a Games-Howell post hoc test for non-homogeneous variances. A value of *p* < 0.05 was considered statistically significant. Variation is expressed as SEM.

## Results

### AZD6738 inhibited phosphorylation of checkpoint kinase 1 and reduced cell proliferation in cultured human conjunctival fibroblasts

**FIGURE 1:**
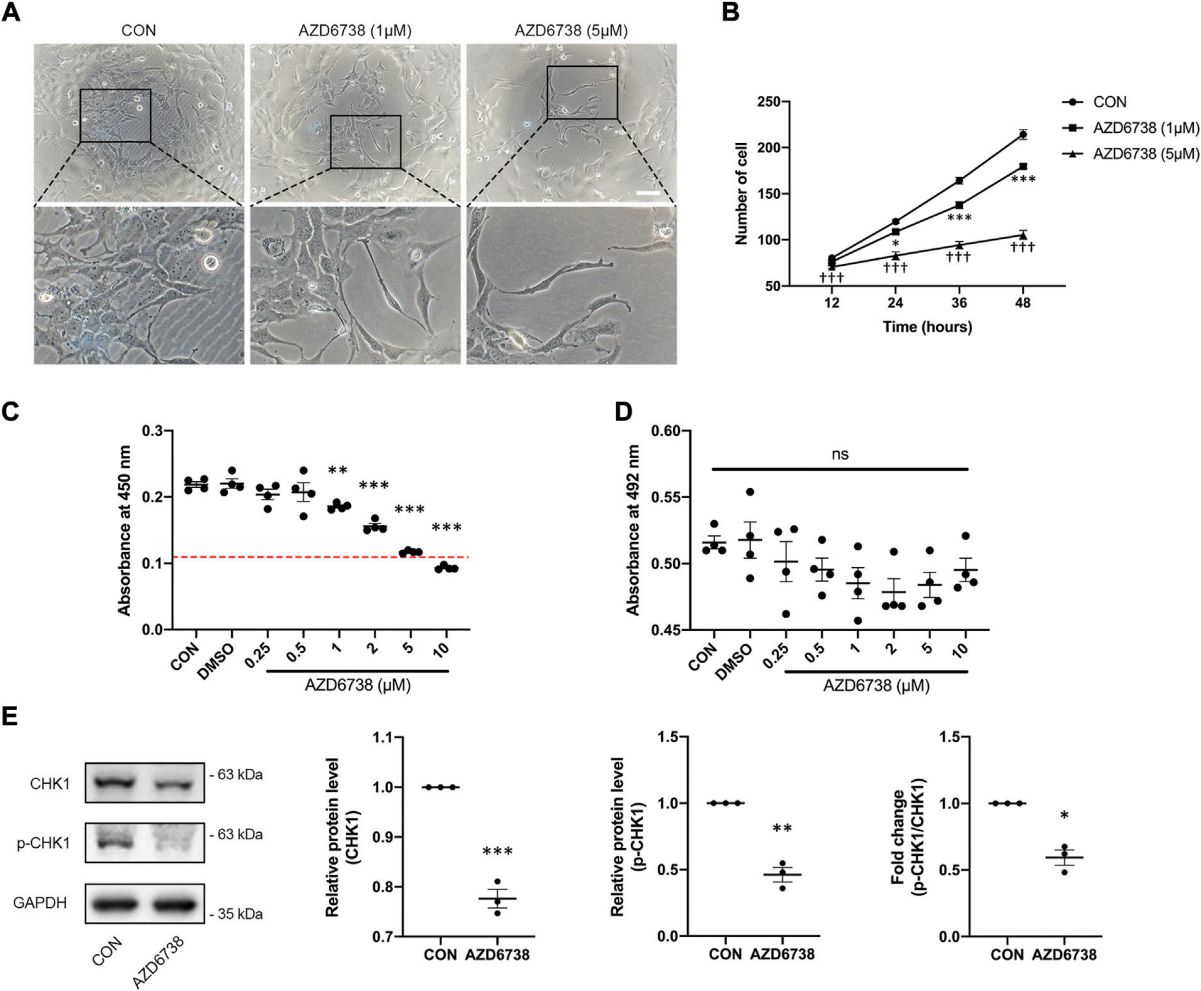
AZD6738 inhibited the expression of CHK1 and reduced the proliferation of HConFs. **(A)** HConFs became round and pseudopodia shrunken with 1 μM AZD6738 treatment, and this phenotype was more obvious on cells with 5 μM AZD6738 treatment. Scale bar: 100 μM. **(B,C)** AZD6738 decreased HConFs viability in a time- and dose-dependent manner. HConFs were treated with 1 and 5 μM of AZD6738 for different hours in **(B)** (*n* = 6), and were treated with different concentrations of AZD6738 for 48 h in **(C)**, the value of IC_50_ was shown by a red dotted line (*n* = 6). **(D)** HConFs were treated with different concentrations of AZD6738, and closed LDH release was detected in the culture medium. **(E)** HConFs were treated with 5 μM of AZD6738 for 48 h, which decreased the level of CHK1 and p-CHK1 and decreased the ratio of p-CHK1/CHK1 (*n* ≥ 3). ^*^ *p* < 0.05, ^**^ *p* < 0.01, and ^***^ *p* < 0.001 versus the blank control group **(B,C,E)**; ^†††^ *p* < 0.001 versus the blank control and AZD6738 (1 μM) group **(B)**. n. s., no significance **(D)**. Results were shown as mean ± SEM.

AZD6738 was a potent inhibitor of ATR kinase activity that can lead to a time- and dose-dependent decrease of cell viability in various cancer cell types (Barnieh et al., 2022; Jo et al., 2022). To verify whether AZD6738 can reduce the proliferation of HConFs in a time- and dose-dependent manner, we determined the cell growth curve of cultured HConFs and the viability of cells treated with 1 and 5 μM AZD6738, and the confirmed IC_50_ of AZD6738 that could be used to establish an *in vitro* model of the inhibited ATR kinase activity of HConFs. There was a decrease in cell proliferation after HConFs were exposed to 1 μM AZD6738 for 24 h, with the shape of cells becoming round and pseudopodia shrunken. As the concentration of AZD6738 was increased to 5 μM, cell proliferation was inhibited earlier to 12 h after exposure (Figures 1A,B). When treated with different concentrations (0.25–10 μM) of AZD6738 for 48 h, the proliferation of HConFs was decreased in a dose-dependent manner (IC_50_ = 7.4 μM; Figure 1C). Moreover, to exclude the effects of drug toxicity from AZD6738 on HConFs, the LDH release was determined by LDH release assay. As shown in Figure 1D, AZD6738 at concentrations up to 10 μM did not increase LDH release in the culture HConFs (Figure 1D). ATR directly mediates the phosphorylation and activity of CHK1. Further, we measured the level of CHK1 and p-CHK1. The results showed that compared to the blank control group, the expression of CHK1 and p-CHK1 and the ratio of p-CHK1/CHK1 was significantly down-regulated following treatment with 5 µM AZD6738 for 48 h (Figure 1E). Accordingly, the concentration of 5 μM AZD6738 was employed for subsequent experiments.

### AZD6738 reduced TGF-β1-induced cell proliferation, migration and collagen utilization in human conjunctival fibroblasts

**FIGURE 2:**
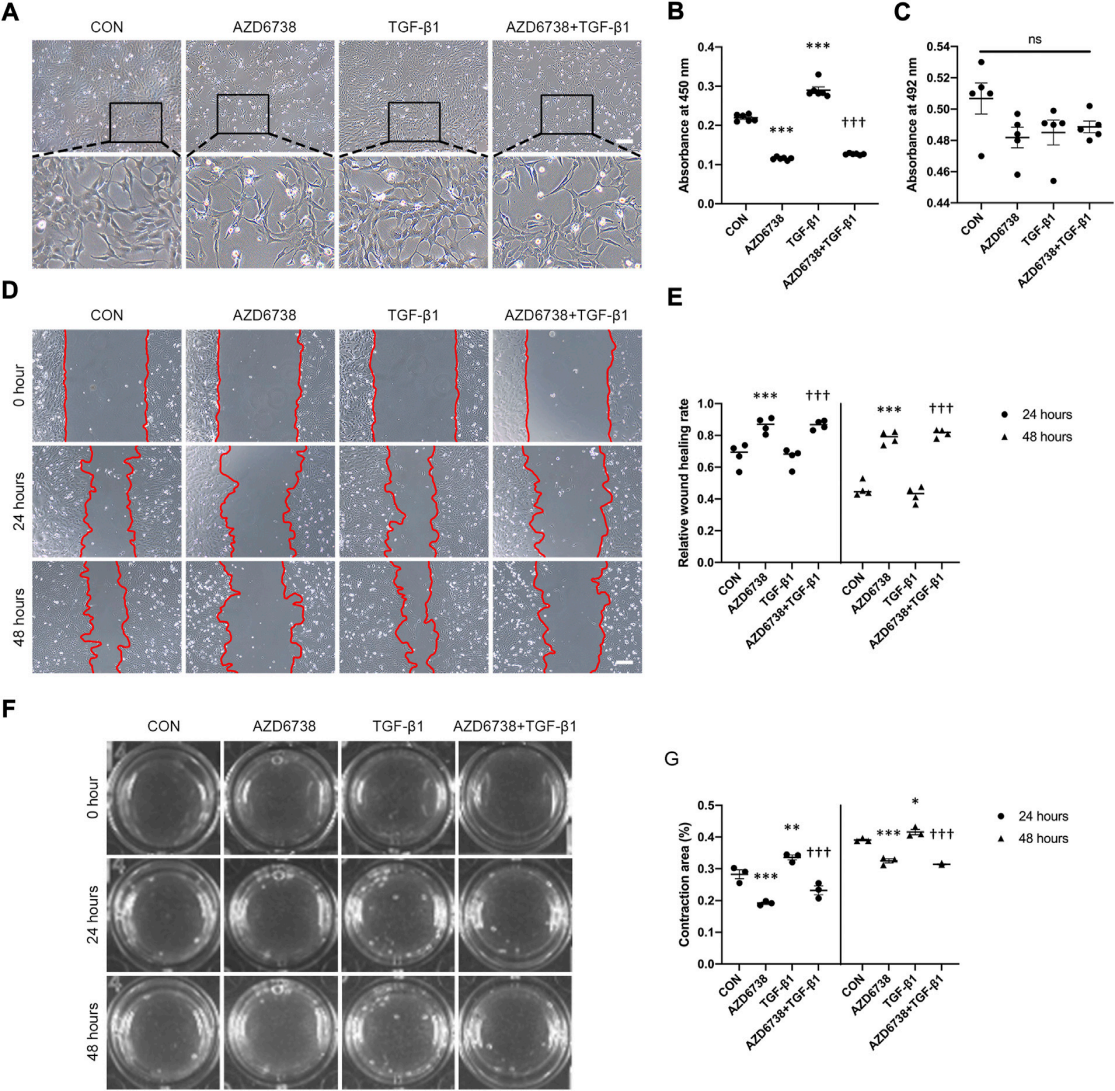
AZD6738 reduced TGF-β1-induced cell proliferation and migration in HConFs. **(A)** HConFs become aggregated and protrude more pseudopodia after TGF-β1 (10 ng/ml) induction, and this phenotype could be inhibited by AZD6738. Scale bar: 200 μM. **(B,C)** HConFs were treated with 5 μM of AZD6738 and 10 ng/ml TGF-β1 for 48 h, the accelerated cell proliferation induced by TGF-β1 was inhibited by AZD6738, and closed LDH release was detected in the culture medium (*n* = 6). (D,E) AZD6738 significantly inhibited the migration of HConFs. Migration assay in HConFs at 0 h, 24 h, and 48 h after treatment, edges of the migrated cells were dotted with red lines (*n* = 4). Scale bar: 200 μM **(D)**. **(F,G)** Representative images and quantitative analysis of gel contraction of HConFs in the four groups at 0 h, 24 h, and 48 h after treatment. The percentage (%) of contraction area was calculated according to the ratio of contracted area compared to the initial area (*n* = 3). ^*^ *p* < 0.05, ^**^ *p* < 0.01, and ^***^ *p* < 0.001 versus the blank control group; ^†††^ *p* < 0.001 versus the TGF-β1 group **(B,E,G)**. n. s., no significance **(C)**. Results were shown as mean ± SEM.

To further investigate the effects of AZD6738 in HConFs, we conducted a TGF-β1-treated model (10 ng/ml) in cultured HConFs, which was a well-established model for inducing cellular proliferation, migration, and fibrosis *in vitro* (Vaamonde-Garcia et al., 2019; Willard et al., 2020; Correia-Sá et al., 2021). After 10 ng/ml TGF-β1 stimulation, HConFs became aggregated and protrude more pseudopodia, which was inhibited by AZD6738 (Figure 2A). CCK-8 assays were performed to detect the cellular proliferation after HConFs were treated with 5 μM of AZD6738 and 10 ng/ml TGF-β1 for 48 h, the result showed an increased proliferation ability in the TGF-β1 group, and the proliferation ability of HConFs was significantly decreased in the 5 µM AZD6738 groups with or without TGF-β1 respectively (Figure 2B). Moreover, the LDH release exhibited no marked increase in HConFs treated with TGF-β1 and/or AZD6738 (Figure 2C). The cellular migration ability was detected by wound healing assay. The rate of cell migration treated with 5 μM AZD6738 was significantly decreased at 24 and 48 h after scratching compared with the cells with or without TGF-β1 respectively. However, compared to the blank control group, the TGF-β1 group did not show an increased migration ability (Figures 2D,E). Additionally, increased HConFs death was observed after cells were treated with 5 μM AZD6738 for 48 h.

The effects of AZD6738 on HConFs-mediated collagen gel contraction with or without TGF-β1 were detected using a three-dimensional (3D) collagen gel system (Figure 2F). Compared with the control group, the mean contraction area (%) of HconFs was significantly increased in the TGF-β1 group while obviously reduced in the AZD6738 group at 24 and 48 h. Moreover, the mean contraction area was decreased over 24 and 48 h of stimulation in the AZD6738+TGF-β1 group compared with the TGF-β1-only group (Figure 2G).

To further verify the molecular mechanisms of AZD6738-reduced cell proliferation and migration, we quantify the changes in protein levels of total CDC2 and C-MYC, two key regulators of cell cycle (Qiao et al., 2006; Hu et al., 2007; Shi et al., 2014; Kim et al., 2015), by using Western Blot. After treatment with 5 μM AZD6738 for 48 h, the protein levels of total CDC2 in HConFs were significantly decreased in the 5 μM AZD6738 group with or without TGF-β1 compared with the blank control and TGF-β1 group (Figure 5B).

Moreover, we verified the morphology and the expression of total CDC2 in HConFs treated with lower concentrations of AZD6738 (0.1 μM), which was verified to against ATR kinase-dependent CHK1 phosphorylation (p-CHK1) in HConFs but not affect the level of CHK1 protein compared to blank control and TGF-β1-only groups (Figure 6B). We confirmed again that HConFs became aggregated and protrude more pseudopodia after 10 ng/ml TGF-β1 stimulation, and similar to the HConFs treated with 1 μM AZD6738, the TGF-β1-treated HConFs become round and pseudopodia shrunken but not affected the proliferation and aggregation after being treated with 0.1 μM AZD6738 for 48 h, while 5 μM AZD6738 reduced the proliferation, aggregation and protruded pseudopodia in the TGF-β1-treated HConFs model (Figure 6A). The results of Western Blot showed that both 0.1 and 5 μM AZD6738 could significantly reduce the expression of CDC2 protein after being treated with different concentrations of AZD6738 and TGF-β1 compared to blank control and TGF-β1 groups (Figure 6B). Additionally, we found that β-Catenin, a key factor for cell growth and adhesion junction (Lillehoj et al., 2007; Peng et al., 2010), was significantly increased in the 0.1 μM AZD6738 group compared to the blank control and TGF-β1-only groups, however, in the 5 μM AZD6738 group, the β-Catenin level was reduced to close to that of the blank control and TGF-β1 groups (Figure 6B).

### AZD6738 caused oxidative damage and regulated apoptosis in human conjunctival fibroblasts by equilibrating the checkpoint kinase 1/*P53* and *PI3K*/*AKT* pathways in a dose-dependent manner

**FIGURE 3:**
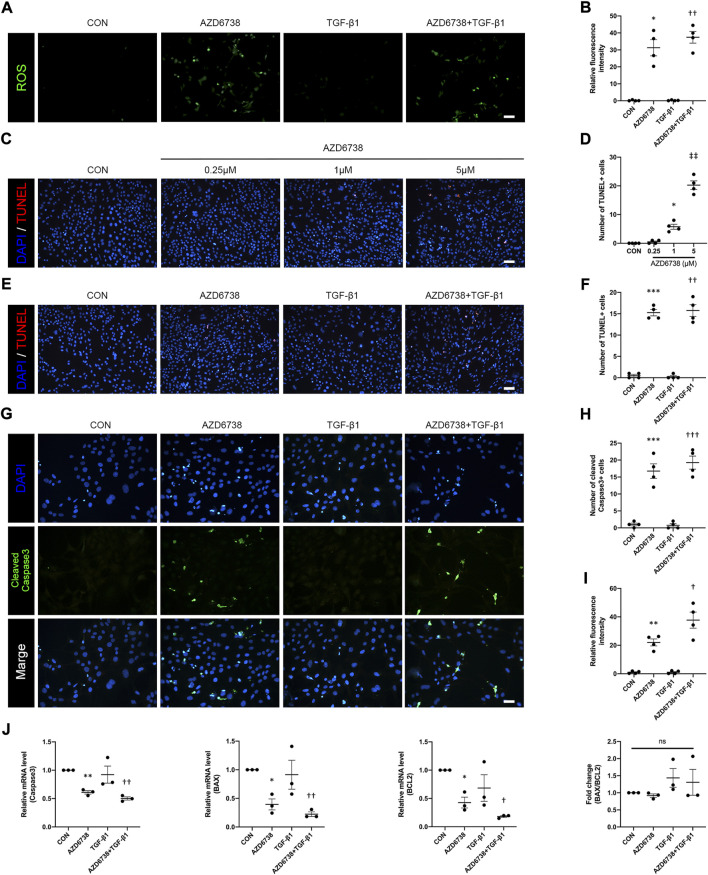
AZD6738 caused oxidative damage and induced apoptosis in HConFs. **(A,B)** AZD6738 increased intracellular ROS generation in HConFs (*n* = 6). Scale bar: 100 μM **(A)**. **(C,D)** AZD6738 induced TUNEL+ cells, and the TUNEL+ cells increased while upregulated the dose of AZD6738 (*n* = 4). Scale bar: 100 μM **(C)**. **(E,F)** AZD6738 also increased the number of TUNEL+ cells in TGF-β1 induced HConFs (*n* = 4). Scale bar: 100 μM **(E)**. **(G–I)** Representative images of HConFs immunofluorescence stained with DAPI (blue) and cleaved Caspase3 (green) in different groups after being treated with 5 μM of AZD6738 and 10 ng/ml TGF-β1 for 48 h **(G)**. The results were quantified as the number of cleaved Caspase3+ cells **(H)** and relative fluorescence intensity normalized to the blank control group (I; *n* = 3), showing that AZD6738 induces the activation of Caspase3. Scale bar: 25 μM **(A)**. **(J)** Quantitative analysis of mRNA expression of *Caspase3*, *BAX* and *BCL2* among the groups after being treated with 5 μM of AZD6738 and 10 ng/ml TGF-β1 for 48 h. The expression of each mRNA level was relative to *GAPDH* and was normalized to the blank control group (*n* ≥ 3). ^*^ *p* < 0.05, ^**^ *p* < 0.01, and ^***^ *p* < 0.001 versus the blank control group; ^†^ *p* < 0.05, ^††^ *p* < 0.01 and ^†††^ *p* < 0.001 versus the TGF-β1 group **(B,D,F,H,I,J)**; ^‡‡^ *p* < 0.01 versus the blank control group and AZD6738 (1 μM) group **(D)**. n. s., no significance **(J)**. Results were shown as mean ± SEM.

To determine whether AZD6738 can induce oxidative stress in HConFs, we measured the intracellular ROS level. The results showed that 5 μM AZD6738 promoted the generation of intracellular ROS, which indicated possible mitochondrial inner membrane damage (Figures 3A,B). It has been reported that AZD6738 can significantly increase apoptosis (Leonard et al., 2019; Nam et al., 2019). TUNEL and immunofluorescence were performed to further verify whether AZD6738 can induce apoptosis in HConFs. We found that AZD6738 started to induce TUNEL+ cells at a concentration of 1 μM, and significantly increased the number of TUNEL+ cells at a concentration of 5 μM, which presented in a dose-dependent manner. However, low concentrations of AZD6738 (0.25 μM) did not induce apoptosis of HConFs (Figures 3C,D). Consistently, in the TGF-β1-treated HConFs model, the TUNEL+ cells increased significantly in the 5 μM AZD6738 group with or without TGF-β1, respectively (Figures 3E,F). The expression of cleaved Caspase3 significantly increased in HConFs treated with 5 μM AZD6738 with or without TGF-β1 compared with that in the blank control and TGF-β1-only groups (Figures 3G–I), which was shown that AZD6738 can induce the activation of Caspase3.

To explore the molecular mechanisms and underlying effects of AZD6738, qPCR was used to quantify the mRNA levels of apoptosis-related factors in HConFs after being treated with 5 μM AZD6738 and 10 ng/ml TGF-β1 for 48 h. The mRNA level of *Caspase3*, *BAX*, *BCL2* and *AKT* in HConFs was significantly decreased, while *P53* was significantly increased in the 5 μM AZD6738 group with or without TGF-β1 compared with the blank control and TGF-β1 groups (Figure 3J and Figure 5B). Interestingly, due to the simultaneously occurring decrease in the levels of *BAX* and *BCL2*, the ratio of *BAX*/*BCL2* did not differ between groups (Figure 3J).

To further clarify the mechanism of AZD6738-induced apoptosis of HConFs, two different concentrations of AZD6738 (0.1 and 5 μM) were set up and used in the TGF-β1-treated HConFs model, and the lower concentration of AZD6738 (0.1 μM) could inhibit CHK1 phosphorylation in HConFs but not reduced the level of CHK1 protein as mentioned above. Similarly, we used qPCR to quantify the changes in mRNA levels of *P53*, *AKT*, *BAX* and *BCL2* in the TGF-β1-treated HConFs model after being treated with 0.1 and 5 μM AZD6738 for 48 h, and we validated the up-regulation of *P53* and the down-regulation of *AKT*, *BAX* and *BCL2* mRNA levels in the TGF-β1 + 5 μM AZD6738 group compared to the blank control and TGF-β1 groups. However, in the TGF-β1 + 0.1 μM AZD6738 group, the mRNA level of *P53* and *BCL2* was significantly increased, while there was no change in the level of *AKT* and *BAX*, compared to the blank control and TGF-β1 groups (Figure 6C).

### AZD6738 reduced TGF-β1-induced myofibroblast activation and extracellular matrix protein synthesis in human conjunctival fibroblasts by inhibiting checkpoint kinase 1*/P53* and *PI3K/AKT* pathways

**FIGURE 4:**
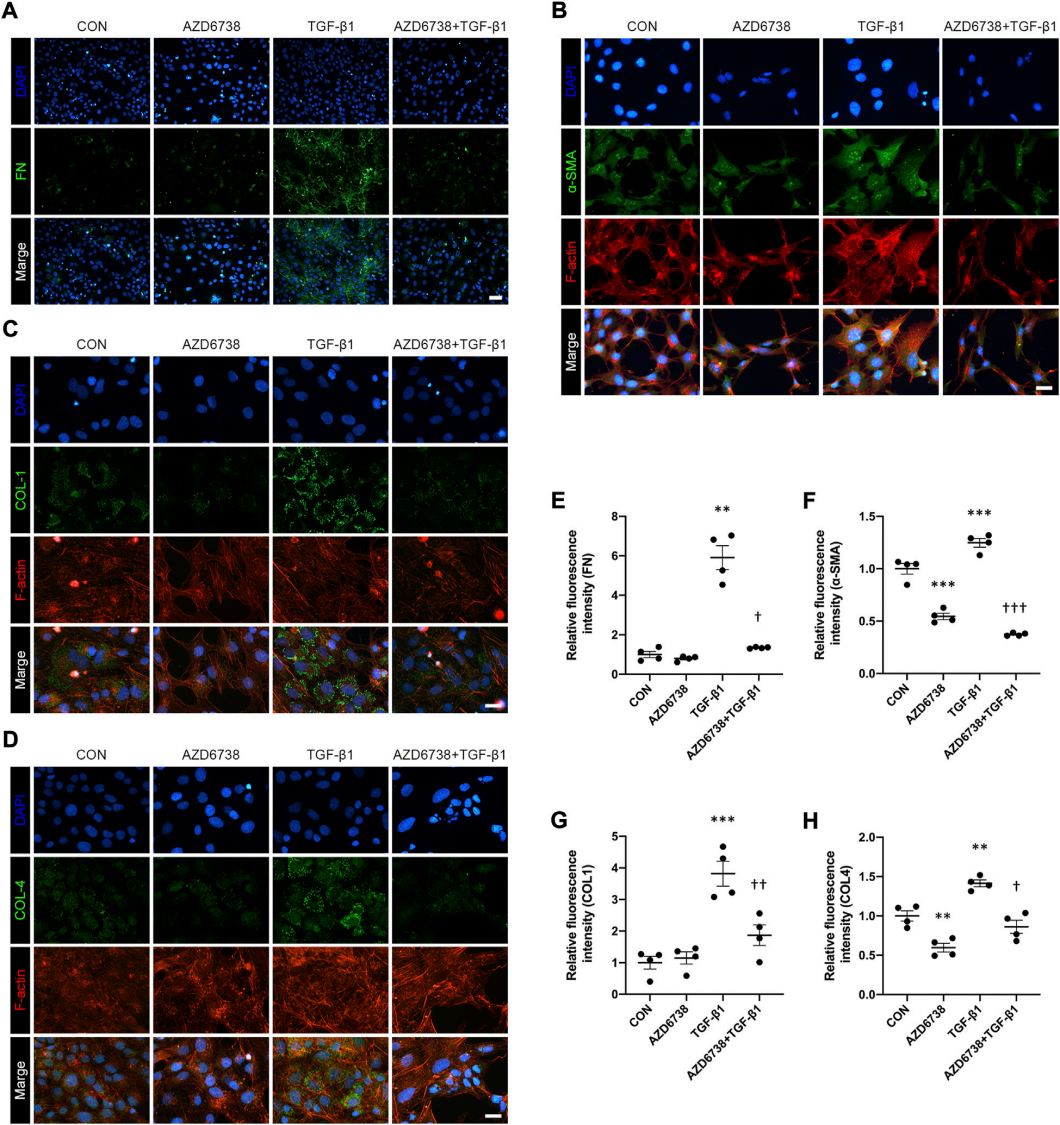
AZD6738 reduced TGF-β1-induced myofibroblast activation and ECM protein synthesis in HConFs. **(A–D)** Representative images of HConFs immunofluorescence stained with DAPI (A, B, G and H; blue), FN **(A)**, α-SMA **(B)**, COL1 **(C)**, COL4 (D; green), and F-actin cytoskeleton (B, C and D; red) in different groups after being treated with 5 μM of AZD6738 and 10 ng/ml TGF-β1 for 48 h, showing the TGF-β1-induced fibrosis was inhibited by AZD6738. Versus the TGF-β1 group, the immunofluorescence of F-actin also showed a decreased expression after being treated with 5 μM AZD6738 for 48 h. Scale bar: 50 μM **(A)** and 25 μM **(B–D)**. **(E–H)** The fluorescence intensity of each group was normalized to blank control group (*n* = 4). ^*^ *p* < 0.05, ^**^ *p* < 0.01, and ^***^ *p* < 0.001 versus the blank control group; ^†^ *p* < 0.05, ^††^ *p* < 0.01 and ^†††^ *p* < 0.001 versus the TGF-β1 group **(E–H)**. Results were shown as mean ± SEM.

CHK1 plays a key role in lung fibrosis (Wu et al., 2022). The TGF-β1-treated HConFs, a model for inducing cellular fibrosis *in vitro* as mentioned above, were used to investigate the antifibrotic effects of AZD6738 in HConFs. We used immunofluorescence to detect the level of fibrosis factors including FN, α-SMA, COL1 and COL4 in HConFs after being treated with 5 μM AZD6738 and 10 ng/ml TGF-β1 for 48 h. The results showed that the fluorescence intensity of FN, α-SMA, COL1 and COL4 was significantly increased in the TGF-β1 group versus the blank control group, and 5 μM AZD6738 significantly inhibited the fluorescence intensity of these fibrotic factors activated by TGF-β1 (Figures 4A–H). Moreover, the immunofluorescence of F-actin also showed a decreased expression after being treated with 5 μM AZD6738 for 48 h compared to the TGF-β1 group (Figures 4B–D).

**FIGURE 5:**
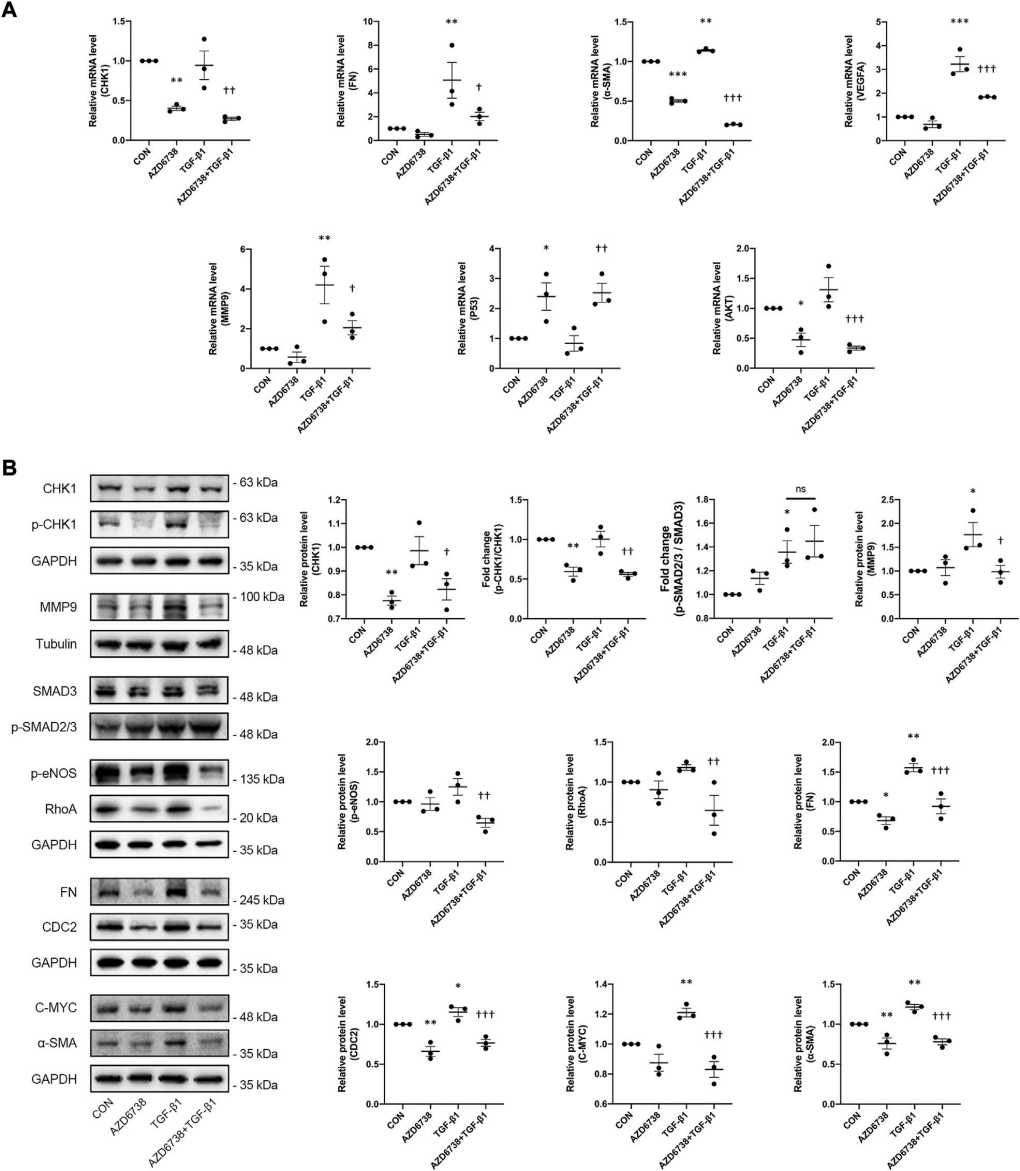
AZD6738 down-regulated the expression of genes related to fibrosis induced by TGF-β1 through *CHK1/P53* and *PI3K/AKT* pathways. **(A)** Quantitative analysis of mRNA expression of *CHK1*, *FN*, *α-SMA*, *VEGFA*, *MMP9*, *P53* and *AKT* among the groups after being treated with 5 μM of AZD6738 and 10 ng/ml TGF-β1 for 48 h. The expression of each mRNA level was relative to *GAPDH* and was normalized to the blank control group (*n* ≥ 3). **(B)** Western blot images and quantitative analysis of CHK1, p-CHK1, FN, α-SMA, MMP9, RhoA, CDC2, p-eNOS, and C-MYC proteins among the groups after being treated with 5 μM of AZD6738 and 10 ng/ml TGF-β1 for 48 h (*n* ≥ 3). GAPDH and α-Tubulin was used as a loading control, and each protein level was normalized to the blank control group (*n* ≥ 3). ^*^ *p* < 0.05, ^**^ *p* < 0.01, and ^***^ *p* < 0.001 versus the blank control group; ^†^ *p* < 0.05, ^††^ *p* < 0.01 and ^†††^ *p* < 0.001 versus the TGF-β1 group **(A,B)**. Results were shown as mean ± SEM.

The effects of AZD6738 on the expression of the profibrotic genes including FN, α-SMA and MMP9 were further tested by qPCR and Western Blot. Compared to the blank control group, AZD6738 significantly decreased the expression of FN at the protein level, and α-SMA at both mRNA and protein levels in cultured HConFs. On the contrary, 10 ng/ml TGF-β1 significantly increased the mRNA and protein levels of FN, α-SMA and MMP9. In the AZD6738 + TGF-β1 group, the TGF-β1-induced high FN, α-SMA and MMP9 level in mRNA and protein were inhibited by AZD6738 (Figures 5A,B). Additionally, factors including RhoA, VEGFA and p-eNOS relative to actin cytoskeleton regulation, angiogenesis and cell proliferation were detected by qPCR and Western Blot. At the mRNA level, the *VEGFA* was significantly increased in the TGF-β1 group compared with the blank control group, and inhibited by AZD6738 in the AZD6738 + TGF-β1 group compared with the TGF-β1 group (Figure 5A). At the protein level, AZD6738 + TGF-β1 group showed a down-regulation of RhoA and p-eNOS compared to the TGF-β1 group, but these two factors had not to be up-regulated in the TGF-β1 group compared with the blank control group. Furthermore, TGF-β1 induced a fibrotic response by phosphorylating SMAD3. However, compared with the TGF-β1-only group, the 5 μM AZD6738+TGF-β1 group did not show significant changes in the ratio of p-SMAD2/3/SMAD3. (Figure 5B).

**FIGURE 6:**
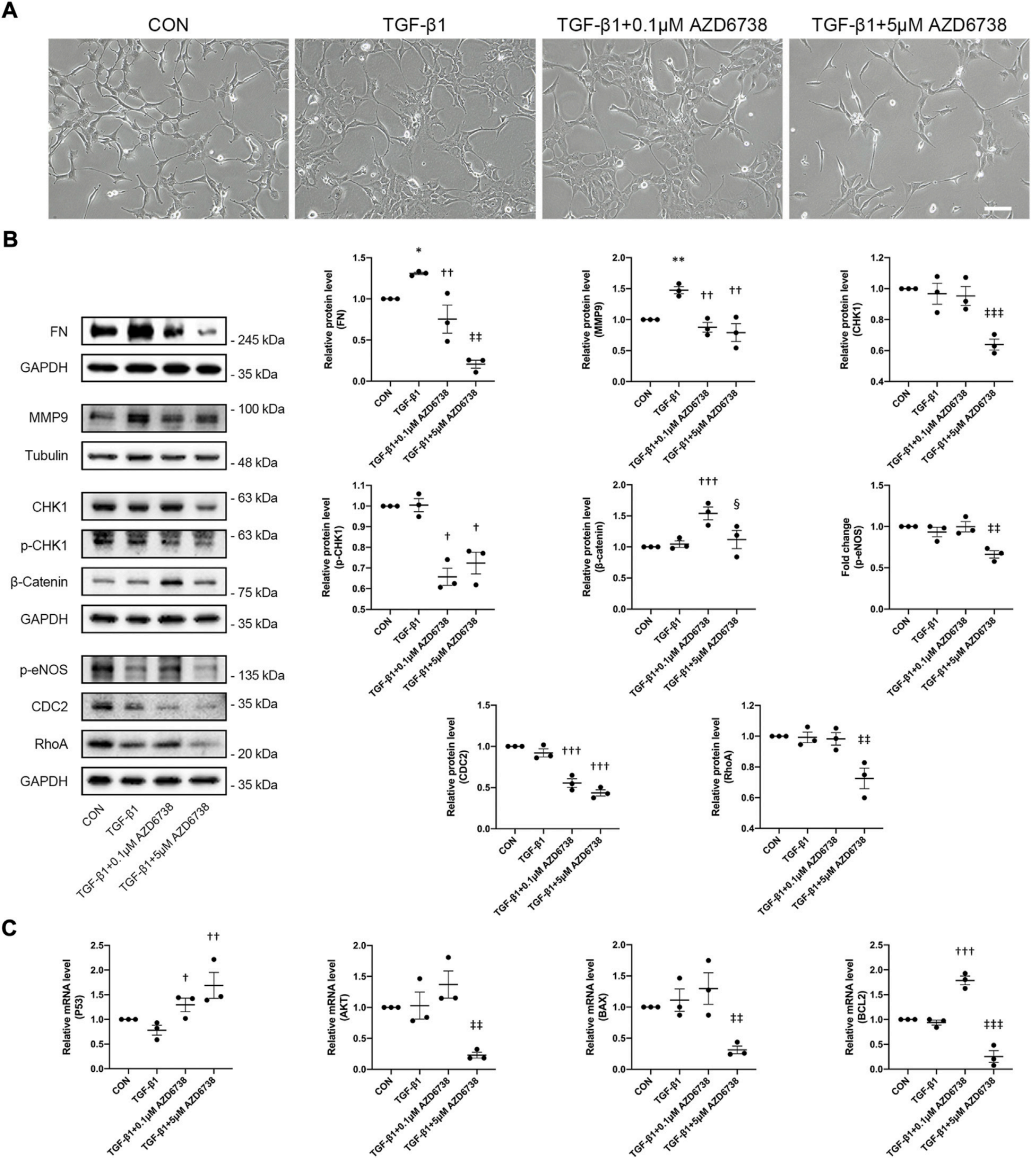
AZD6738 regulated *CHK1/P53* and *PI3K/AKT* pathways and the expression of genes related to fibrosis and apoptosis in a dose-dependent manner. **(A)** After treatment for 48 h, 0.1 μM AZD6738 inhibited the protruded pseudopodia of HConFs induced by TGF-β1 (10 ng/ml) but not affected the proliferation and aggregation, while 5 μM AZD6738 reduced all the proliferation, aggregation and protruded pseudopodia induced by TGF-β1. Scale bar: 200 μM. **(B)** Western blot images and quantitative analysis of FN, MMP9, CHK1, p-CHK1, p-eNOS, RhoA, β-Catenin, and CDC2 proteins among the groups after being treated with 0.1 or 5 μM of AZD6738 and 10 ng/ml TGF-β1 for 48 h (*n* ≥ 3). GAPDH and α-Tubulin was used as a loading control, and each protein level was normalized to the blank control group (*n* ≥ 3). **(C)** Quantitative analysis of mRNA expression of *P53*, *AKT*, *BAX* and *BCL2* among the groups after being treated with 0.1 or 5 μM of AZD6738 and 10 ng/ml TGF-β1 for 48 h. The expression of each mRNA level was relative to *GAPDH* and was normalized to the blank control group (*n* ≥ 3). ^*^ *p* < 0.05, ^**^ *p* < 0.01, and ^***^ *p* < 0.001 versus the blank control group; ^†^ *p* < 0.05, ^††^ *p* < 0.01 and ^†††^ *p* < 0.001 versus the TGF-β1 group; ^‡‡^ *p* < 0.01 and ^‡‡‡^ *p* < 0.001 versus the TGF-β1 group and the TGF-β1 + 0.1 μM AZD6738 group; ^§^ *p* < 0.05 versus the TGF-β1 + 0.1 μM AZD6738 group **(B,C)**. Results were shown as mean ± SEM.

As mentioned above, 5 μM AZD6738 inhibited both ATR kinase and PI3Kδ and affected the *CHK1*/*P53* and *PI3K*/*AKT* pathways at the same time. To further explore the mechanism of AZD6738 inhibiting HConFs fibrosis, we also used high (5 μM) and low (0.1 μM) concentrations of AZD6738 to treat the TGF-β1-activated HConFs. The protein level of FN and MMP9 were quantified by Western Blot. The result showed that the FN level was decreased in the TGF-β1 + 0.1 μM AZD6738 group compared to the TGF-β1 group, and was also significantly decreased in the TGF-β1 + 5 μM AZD6738 group compared to the TGF-β1 + 0.1 μM AZD6738 group, which presented in a dose-dependent manner (Figure 6B). Notably, the protein level of MMP9 was significantly decreased both in the TGF-β1 + 0.1 μM AZD6738 and TGF-β1 + 5 μM AZD6738 group compared to the TGF-β1 group, but showed no distinction between the two groups (Figure 6B). Furthermore, there were no distinction in RhoA and p-eNOS protein levels among the blank control, TGF-β1 and TGF-β1 + 0.1 μM AZD6738 groups, while these protein levels were significantly decreased in the TGF-β1 + 5 μM AZD6738 group versus the other three groups (Figure 6B).

## Discussion

In this study, we revealed the effects and detailed mechanism of AZD6738 on cultured HConFs *in vitro* and confirmed the anti-fibrotic efficacy of AZD6738 in the TGF-β1-induced HConFs model, providing basic evidence for further studies on anti-subconjunctival fibrosis.

Oncogenic mutations lead to errors in DNA replication that cannot be repaired. Therefore, ATR inhibitors, a protein considered to be responsible for DNA repair, are of wide interest in the antitumor field. In several published clinical trials, patients with advanced bowel cancer and ovarian cancer benefited from ATR inhibitor therapy (Konstantinopoulos et al., 2020; Yap et al., 2020). Notably, AZD6738 could inhibits *PI3Kδ* and affect the *PI3K*/*AKT* pathway at high doses (IC_50_ = 6.8 μM) which was close to the IC_50_ we measured affecting the proliferation of HconFs. This may indicate the effect of AZD6738 on the cell proliferation was not merely by inhibiting the activity of ATR kinase. Therefore, follow-up experiments would further verify the detailed mechanism of the effect of AZD6738 on HConFs. Indeed, we apply ATR inhibitor AZD6738 to ocular cells for the first time and found that it affects HConFs through a dual pharmacological mechanism of *CHK1*/*P53* and *PI3K*/*AKT* pathways, which differs from previously observed effects of AZD6738 on several cancer cells.

CHK1 plays a key role in DNA replication stress response or DNA damage repair (Zhao et al., 2002; Chen et al., 2003). It is of great significance to the regulation of phosphorylated CHK1. Consistent with our findings, a recent report described that AZD6738 reduced CHK1 phosphorylation in sensitive cell lines. However, these AZD6738-sensitive cell lines all exhibited low expression of P53 (Nam et al., 2019). Our study found that AZD6738 was also able to reduce the phosphorylation of CHK1 in normal HConFs and the level of total CHK1 and phosphorylated CHK1 changed with the dose of AZD6738. HConFs responded to a low concentration of AZD6738 (0.1 μM) by only reducing the level of phosphorylated CHK1. As the concentration of AZD6738 increased to 5 μM, the level of total CHK1 also decreased significantly, while the mRNA expression of *P53* was up-regulated with an increasing concentration of AZD6738. Similarly, the expression of *AKT* also showed a significant correlation with the concentration of AZD6738. This result shows that AZD6738 may have different mechanisms of action on HConFs at different concentrations. We propose a potential mechanism by which AZD6738 regulates the proliferation, migration, myofibroblast activation and ECM protein synthesis of HConFs through further observation of this phenomenon.

Although research showed the ability of AZD6738 to induce apoptosis (Nam et al., 2019; Leonard et al., 2019; Barnieh et al., 2022), our study founds that low concentrations of AZD6738 (0.1 μM) seems to protect HConFs from apoptosis. We first used 5 μM AZD6738 to treat HConFs and identified possible oxidative stress and mitochondrial membrane damage by detecting intracellular ROS generation in HConFs. We confirmed that HConFs underwent apoptosis in response to the treatment of 5 μM AZD6738 in further TUNEL assays. To further explore whether mitochondrial membrane permeability is altered in the presence of AZD6738, we evaluated the expression levels of *BAX* and *BCL2*, two mitochondrial membrane integrins that play a critical role in the apoptotic pathway (Eguchi et al., 1992; Schmitt er al., 2000; Ruvolo et al., 2001). Interestingly, unlike the classical mitochondrial pathway of apoptosis (Feng et al., 2008; Kakarla et al., 2010), 5 μM AZD6738 down-regulated both *BAX* and *BCL2* levels. Moreover, we used a low concentration of AZD6738 (0.1 μM), which caused down-regulation of phosphorylated CHK1 expression and up-regulation of *P53* expression, respectively, but did not change the expression of *AKT* and induce apoptosis in HConFs. The result showed that the expression of *BAX* did not change under the influence of 0.1 μM AZD6738, while that of *BCL2* was significantly up-regulated, which is considered improving cell survival and inhibit apoptosis.

Based on this result, we propose that AZD6738 controls the survival and apoptosis of HConFs in a dose-dependent manner by balancing the *CHK1*/*P53* and *PI3K*/*AKT* pathways. Low-dose AZD6738 inhibited the phosphorylation of CHK1 and further inhibited the phosphorylation of P53 and lost its function, and the non-functional P53 up-regulated the mRNA level of *P53* in the form of negative feedback. On the one hand, the non-functional P53 cannot induce the expression of *BAX* in DNA, and up-regulates the expression of *BCL2* through pathways like *P53*/*Siva1* on the other hand to protect HConFs from apoptosis (Du et al., 2009; Wang et al., 2013). When the concentration of AZD6738 was increased, the *PI3K*/*AKT* pathway was inhibited, the expression of *BCL2* was down-regulated through the *AKT*/*BAD* and *AKT*/*CREB* pathways (Liu et al., 2020; Huang et al., 2021; Xu et al., 2021), and the apoptosis of HConFs was induced with the changed *BAX*/*BCL2* ratio.

In recent years, CHK1, a major substrate of ATR as mentioned above, is proved to involve in the accumulation of activated myofibroblasts and secrete elevated levels of growth factors that stimulate cell proliferation and ECM production in both autocrine and paracrine manners, which underscores the pivotal role of CHK1 in myofibroblast activation (Wu et al., 2022). However, the mechanism by altered *ATR*/*CHK1* expression affects the activation of myofibroblasts and the synthesis of ECM protein is still unclear. We have successfully used a well-established TGF-β1-induced rabbit tenon’s fibroblasts (RTFs) *in vitro* model in a previous study, which can increase RTFs proliferation, migration, collagen utilization, ECM protein synthesis and myofibroblast activation (Lan et al., 2021). In this study, we used the TGF-β1-treated HConFs model and observed whether AZD6738 inhibited TGF-β1-induced myofibroblast activation and ECM protein synthesis in HConFs.

Our findings suggest that AZD6738 has significant efficacy in reducing myofibroblast activation as indicated by the synthesis of α-SMA and ECM-related proteins such as FN, COL1 and COL4 in a TGF-β1-induced HConFs model. By comparing the antifibrotic effects of low (0.1 μM) and high (0.5 μM) AZD6738 on HConFs, we found that low doseAZD6738 showed good antifibrotic effects, including reversal of TGF-β1-induced elevation of FN and MMP9, and high-dose AZD6738 further downregulated FN expression. This suggests that AZD6738 regulates the level of MMP9 through the *CHK1*/*P53* pathway and jointly regulates the expression of FN in HConFs through the *CHK1*/*P53* and *PI3K*/*AKT* pathways, while the expression level of MMP9 is close to the expression level after low-dose AZD6738 treatment. Different from *in vivo*, MMP9 is secreted from intracellular to extracellular in the form of the zymogen, and the activation of MMP9 *in vitro* needs to react with organic Mercury preparations (Yang et al., 2010; Khan et al., 2017). Therefore, the down-regulation of MMP9 *in vitro* under the action of different doses of AZD6738 verified the dual pharmacological mechanism of AZD6738 against fibrosis, which was caused by the negative feedback caused by the down-regulation of the target proteins like COL4 and COL5 (Tschesche et al., 1992). Further, we revealed that AZD6738 reduces the expression of *VEGFA* in HConFs possible by down-regulating the phosphorylation level of eNOS through *AKT*/*eNOS* pathway (Guo et al., 2021; Ninchoji et al., 2021), which shows the potential of AZD6738 in the treatment of neovascular eye diseases. Although TGF-β1 did not induce the up-regulation of RhoA level in HConFs in our research, the administration of high-dose AZD6738 still reduced the expression of RhoA to a certain extent, which indicating the potential effect of AZD6738 on the cytoskeleton.

In our research, the phosphorylation level of SMAD3 did not change in HConFs after AZD6738 intervention. The addition of TGF-β1 also did not significantly affect the expression of CHK1 and AKT and change the phosphorylation level of CHK1. Our results indicated that TGF-β1 induced the occurrence and development of fibrotic response through the canonical *TGF-β1/SMAD2/3* signal pathway, and AZD6738 did not directly affect the phosphorylation of SMAD3. P53, the substrate of CHK1, plays an important role in the fibrosis process. P53-SMAD3 transcriptional cooperation downstream of TGF-β1 orchestrates induction of fibrotic factors, ECM accumulation, and pathogenic renal cell communication (Overstreet et al., 2022). Decreased PI3K expression and AKT phosphorylation can delay renal fibrosis through the *PI3K/AKT/mTOR* signaling cascade in HN rat renal hypertrophy model (Zhou et al., 2022). Changes in the expression of P53 and AKT were also associated with liver fibrosis (Zhang et al., 2022). We interpret the underlying molecular mechanism by which AZD6738 inhibited TGF-β1-induced fibrotic response in HConFs based on the existing evidence mentioned above.

The safety of ophthalmic medication is one of the major concerns. Although high-dose AZD6738-induced apoptosis of HConFs is beneficial to anti-subconjunctival scarring and the LDH release assay also proved the safety of AZD6738 on HConFs, the possibility of non-specific cytotoxicity to other cells in the eye cannot be ignored. Compared with other antiproliferative agents that have been applied in ophthalmic at present, the special dual pharmacological mechanism of AZD6738 enables it to work at a low dose without affecting cell proliferation, not inducing or even fighting apoptosis. We found that the expression of total CDC2 was significantly down-regulated after being treated with a low concentration (0.1 μM) of AZD6738 due to inhibition of CHK1 phosphorylation, which indicated the dephosphorylated and functional of CDC2. However, in the cell proliferation assay, low concentrations of AZD6738 showed no inhibitory effects on proliferation of HConFs, indicating the existence of a possible cell cycle checkpoint compensation mechanism. Anyway, the safety of AZD6738 in ophthalmic applications needs to be evaluated in further *in vivo* experiments. Furthermore, AZD6738 can enhance the sensitivity of cancer cells to 5-FU (Suzuki et al., 2022), which provides the possibility of AZD6738/5-FU combination to fight subconjunctival scarring in the eye, implying lower dosage, higher safety, and better efficacy.

AZD6738 can simultaneously affect both *CHK1/P53* and *PI3K/AKT* signaling pathways. Although the results in this study suggest that AZD6738 affects HConFs in a dual pharmacological mechanism at different concentrations, more associated signaling pathway inhibitors such as specific CHK1 kinase or PI3K inhibitors should be used in future studies to determine whether other inhibitors can phenocopy these results, which will suggest that they are generic features of inhibition of these pathways. The underlying signaling pathway for the anti-fibrotic effect by inhibiting CHK1 remains an interesting research direction. In addition, this study was performed *in vitro*, preclinical models will be used in future studies to further evaluate the anti-fibrotic effects of AZD6738 *in vivo*.

In conclusion, our results indicate that AZD6738 affects the survival and apoptosis of HConFs by regulating the *CHK1*/*P53* and *PI3K*/*AKT* pathways, and inhibits TGF-β1-induced fibrosis including myofibroblast activation and relative ECM protein synthesis, which demonstrates the anti-fibrotic response efficacy of AZD6738 in cultured HConFs *in vitro* and may become a potential therapeutic option for anti-subconjunctival scarring after trabeculectomy.

## Data Availability

The original contributions presented in the study are included in the article, further inquiries can be directed to the corresponding authors.

## References

[B1] Addicks E. M. Quigley H. A. Green W. R. Robin A. L. (1983). Histologic characteristics of filtering blebs in glaucomatous eyes. Arch. Ophthalmol. 101 (5), 795–798. 10.1001/archopht.1983.01040010795021 6847472

[B2] Barnieh F. M. Ribeiro M. G. Garland H. Loadman P. M. Falconer R. A. (2022). Targeted delivery of a colchicine analogue provides synergy with ATR inhibition in cancer cells. Biochem. Pharmacol. 201, 115095. 10.1016/j.bcp.2022.115095 35598808

[B3] Bell K. de Padua S. B. B. Mofokeng M. Montesano G. Nongpiur M. E. Marti M. V. (2021). Learning from the past: Mitomycin C use in trabeculectomy and its application in bleb-forming minimally invasive glaucoma surgery. Surv. Ophthalmol. 66 (1), 109–123. 10.1016/j.survophthal.2020.05.005 32450159

[B4] Cabourne E. Clarke J. C. K. Schlottmann P. G. Evans J. R. (2015). Mitomycin C versus 5-Fluorouracil for wound healing in glaucoma surgery. Cochrane Database Syst. Rev. 11, CD006259. 10.1002/14651858.CD006259.pub2 PMC8763343 26545176

[B5] Cairns J. E. (1968). Trabeculectomy. Am. J. Ophthalmol. 66 (4), 673–679. 10.1016/0002-9394(68)91288-9 4891876

[B6] Chen M. Ryan C. E. Piwnica-Worms H. (2003). Chk1 kinase negatively regulates mitotic function of Cdc25A phosphatase through 14-3-3 binding. Mol. Cell. Biol. 23 (21), 7488–7497. 10.1128/MCB.23.21.7488-7497.2003 14559997 PMC207598

[B7] Correia-Sá I. B. Carvalho C. M. Serrão P. V. Machado V. A. Carvalho S. O. Marques M. (2021). AM251, a cannabinoid receptor 1 antagonist, prevents human fibroblasts differentiation and collagen deposition induced by TGF-β - an *in vitro* study. Eur. J. Pharmacol. 892, 173738. 10.1016/j.ejphar.2020.173738 33220269

[B8] Davis B. M. Crawley L. Pahlitzsch M. Javaid F. Cordeiro M. (2016). Glaucoma: The retina and beyond. Acta Neuropathol. 132 (6), 807–826. 10.1007/s00401-016-1609-2 27544758 PMC5106492

[B9] Du W. Jiang P. Li N. Mei Y. Wang X. Wen L. (2009). Suppression of p53 activity by Siva1. Cell Death Differ. 16 (11), 1493–1504. 10.1038/cdd.2009.89 19590512 PMC2883715

[B10] Eguchi Y. Ewert D. L. Tsujimoto Y. (1992). Isolation and characterization of the chicken bcl-2 gene: Expression in a variety of tissues including lymphoid and neuronal organs in adult and embryo. Nucleic Acids Res. 20 (16), 4187–4192. 10.1093/nar/20.16.4187 1508712 PMC334124

[B11] Eldaly M. A. Bunce C. Elsheikha O. Z. Wormald R. (2014). Non-penetrating filtration surgery versus trabeculectomy for open-angle glaucoma. Cochrane Database Syst. Rev. 2, CD007059. 10.1002/14651858.CD007059.pub2 PMC11329888 24532137

[B12] Feijoo C. Hall-Jackson C. Wu R. Jenkins D. Leitch J. Gilbert D. M. (2001). Activation of mammalian Chk1 during DNA replication arrest: A role for Chk1 in the intra-S phase checkpoint monitoring replication origin firing. J. Cell Biol. 154 (5), 913–923. 10.1083/jcb.200104099 11535615 PMC1255922

[B13] Feng P. Li T. Guan Z. Franklin R. B. Costello L. C. (2008). The involvement of Bax in zinc-induced mitochondrial apoptogenesis in malignant prostate cells. Mol. Cancer 7, 25. 10.1186/1476-4598-7-25 18331646 PMC2329666

[B14] Franks W. A. Hitchings R. A. (1991). Complications of 5--fluorouracil after trabeculectomy. Eye 5, 385–389. 10.1038/eye.1991.63 1743353

[B15] Gedde S. J. Feuer W. J. Shi W. Lim K. S. Barton K. Goyal S. (2018). Treatment outcomes in the primary tube versus trabeculectomy study after 1 Year of follow-up. Ophthalmology 125 (5), 650–663. 10.1016/j.ophtha.2018.02.003 29477688

[B16] Green E. Wilkins M. Bunce C. Wormald R. (2014). 5-Fluorouracil for glaucoma surgery. Cochrane Database Syst. Rev. 2, CD001132. 10.1002/14651858.CD001132.pub2 PMC10558100 24554410

[B17] Guo X. Chen M. Cao L. Hu Y. Li X. Zhang Q. (2021). Cancer-associated fibroblasts promote migration and invasion of non-small cell lung cancer cells *via* miR-101-3p mediated VEGFA secretion and AKT/eNOS pathway. Front. Cell Dev. Biol. 9, 764151. 10.3389/fcell.2021.764151 34977016 PMC8716726

[B18] Heffernan T. P. Simpson D. A. Frank A. R. Heinloth A. N. Paules R. S. Cordeiro-Stone M. (2002). An ATR- and Chk1-dependent S checkpoint inhibits replicon initiation following UVC-induced DNA damage. Mol. Cell. Biol. 22 (24), 8552–8561. 10.1128/MCB.22.24.8552-8561.2002 12446774 PMC139882

[B19] Hu X. Cui D. Moscinski L. C. Zhang X. Maccachero V. Zuckerman K. S. (2007). TGFbeta regulates the expression and activities of G2 checkpoint kinases in human myeloid leukemia cells. Cytokine 37 (2), 155–162. 10.1016/j.cyto.2007.03.009 17459720

[B20] Huang J. Yang J. Zou X. Zuo S. Wang J. Cheng J. (2021). Ginkgolide B promotes oligodendrocyte precursor cell differentiation and survival via Akt/CREB/bcl-2 signaling pathway after white matter lesion. Exp. Biol. Med. 246 (10), 1198–1209. 10.1177/1535370221989955 PMC8142115 33557607

[B21] Jo U. Murai Y. Agama K. K. Sun Y. Saha L. K. Yang X. (2022). TOP1-DNA trapping by exatecan and combination therapy with ATR inhibitor. Mol. Cancer Ther. 21 (7), 1090–1102. 10.1158/1535-7163.MCT-21-1000 35439320 PMC9256811

[B22] Kakarla S. K. Fannin J. C. Keshavarzian S. Katta A. Paturi S. Nalabotu S. K. (2010). Chronic acetaminophen attenuates age-associated increases in cardiac ROS and apoptosis in the Fischer Brown Norway rat. Basic Res. Cardiol. 105 (4), 535–544. 10.1007/s00395-010-0094-3 20407780

[B23] Khan H. Singh R. D. Tiwari R. Gangopadhyay S. Roy S. K. Singh D. (2017). Mercury exposure induces cytoskeleton disruption and loss of renal function through epigenetic modulation of MMP9 expression. Toxicology 386, 28–39. 10.1016/j.tox.2017.05.006 28526320

[B24] Kim H. Min A. Im S. Jang H. Lee K. H. Lau A. (2017). Anti-tumor activity of the ATR inhibitor AZD6738 in HER2 positive breast cancer cells. Int. J. Cancer 140 (1), 109–119. 10.1002/ijc.30373 27501113

[B25] Kim J. Cho Y. Park J. (2015). The nucleolar protein GLTSCR2 is an upstream negative regulator of the oncogenic nucleophosmin-MYC Axis. Am. J. Pathol. 185 (7), 2061–2068. 10.1016/j.ajpath.2015.03.016 25956029

[B26] King A. J. Shah A. Nikita E. Hu K. Mulvaney C. A. Stead R. (2018). Subconjunctival draining minimally-invasive glaucoma devices for medically uncontrolled glaucoma. Cochrane Database Syst. Rev. 12, CD012742. 10.1002/14651858.CD012742.pub2 30554418 PMC6517205

[B27] Kitazawa Y. Kawase K. Matsushita H. Minobe M. (1991). Trabeculectomy with mitomycin. A comparative study with fluorouracil. Arch. Ophthalmol. 109 (12), 1693–1698. 10.1001/archopht.1991.01080120077030 1841578

[B28] Konstantinopoulos P. A. Cheng S. Wahner H. A. E. Penson R. T. Schumer S. T. Doyle L. A. (2020). Berzosertib plus gemcitabine versus gemcitabine alone in platinum-resistant high-grade serous ovarian cancer: A multicentre, open-label, randomised, phase 2 trial. Lancet. Oncol. 21 (7), 957–968. 10.1016/S1470-2045(20)30180-7 32553118 PMC8023719

[B29] Lan C. Tan J. Tang L. Liu G. Huang L. Luo X. (2021). Forkhead domain inhibitory-6 attenuates subconjunctival fibrosis in rabbit model with trabeculectomy. Exp. Eye Res. 210, 108725. 10.1016/j.exer.2021.108725 34375589

[B30] Leonard B. C. Lee E. D. Bhola N. E. Li H. Sogaard K. K. Bakkenist C. J. (2019). ATR inhibition sensitizes HPV- and HPV+ head and neck squamous cell carcinoma to cisplatin. Oral Oncol. 95, 35–42. 10.1016/j.oraloncology.2019.05.028 31345392 PMC6827881

[B31] Lillehoj E. P. Lu W. Kiser T. Goldblum S. E. Kim K. C. (2007). MUC1 inhibits cell proliferation by a beta-catenin-dependent mechanism. Biochim. Biophys. Acta 1773 (7), 1028–1038. 10.1016/j.bbamcr.2007.04.009 17524503 PMC2349984

[B32] Liu C. Chen K. Wang H. Zhang Y. Duan X. Xue Y. (2020). Gastrin attenuates renal ischemia/reperfusion injury by a PI3K/Akt/Bad-Mediated anti-apoptosis signaling. Front. Pharmacol. 11, 540479. 10.3389/fphar.2020.540479 33343341 PMC7740972

[B33] Lusthaus J. Goldberg I. (2019). Current management of glaucoma. Med. J. Aust. 210 (4), 180–187. 10.5694/mja2.50020 30767238

[B34] Mathew D. J. Buys Y. M. (2020). Minimally invasive glaucoma surgery: A critical appraisal of the literature. Annu. Rev. Vis. Sci. 6, 47–89. 10.1146/annurev-vision-121219-081737 32936738

[B35] Mearza A. A. Aslanides I. M. (2007). Uses and complications of mitomycin C in ophthalmology. Expert Opin. Drug Saf. 6 (1), 27–32. 10.1517/14740338.6.1.27 17181449

[B36] Nam A. Jin M. H. Park J. E. Bang J. Oh D. Bang Y. (2019). Therapeutic targeting of the DNA damage response using an ATR inhibitor in biliary tract cancer. Cancer Res. Treat. 51 (3), 1167–1179. 10.4143/crt.2018.526 30514066 PMC6639230

[B37] NikhalaShree S. Karthikkeyan G. George R. Shantha B. Vijaya L. Ratra V. (2019). Lowered decorin with aberrant extracellular matrix remodeling in aqueous humor and tenon's tissue from primary glaucoma patients. Invest. Ophthalmol. Vis. Sci. 60 (14), 4661–4669. 10.1167/iovs.19-27091 31725165

[B38] Ninchoji T. Love D. T. Smith R. O. Hedlund M. Vestweber D. Sessa W. C. (2021). eNOS-induced vascular barrier disruption in retinopathy by c-Src activation and tyrosine phosphorylation of VE-cadherin. Elife 10, e64944. 10.7554/eLife.64944 33908348 PMC8087444

[B39] Overstreet J. M. Gifford C. C. Tang J. Higgins P. J. Samarakoon R. (2022). Emerging role of tumor suppressor p53 in acute and chronic kidney diseases. Cell. Mol. Life Sci. 79 (9), 474. 10.1007/s00018-022-04505-w 35941392 PMC11072039

[B40] Peng X. Cuff L. E. Lawton C. D. DeMali K. A. (2010). Vinculin regulates cell-surface E-cadherin expression by binding to beta-catenin. J. Cell Sci. 123, 567–577. 10.1242/jcs.056432 20086044 PMC2818194

[B41] Qiao M. Shapiro P. Fosbrink M. Rus H. Kumar R. Passaniti A. (2006). Cell cycle-dependent phosphorylation of the RUNX2 transcription factor by cdc2 regulates endothelial cell proliferation. J. Biol. Chem. 281 (11), 7118–7128. 10.1074/jbc.M508162200 16407259

[B42] Quigley H. A. Broman A. T. (2006). The number of people with glaucoma worldwide in 2010 and 2020. Br. J. Ophthalmol. 90 (3), 262–267. 10.1136/bjo.2005.081224 16488940 PMC1856963

[B43] Ruvolo P. P. Deng X. May W. S. (2001). Phosphorylation of Bcl2 and regulation of apoptosis. Leukemia 15 (4), 515–522. 10.1038/sj.leu.2402090 11368354

[B44] Santos J. M. Camões S. P. Filipe E. Cipriano M. Barcia R. N. Filipe M. (2015). Three-dimensional spheroid cell culture of umbilical cord tissue-derived mesenchymal stromal cells leads to enhanced paracrine induction of wound healing. Stem Cell Res. Ther. 6, 90. 10.1186/s13287-015-0082-5 25956381 PMC4448539

[B45] Schlunck G. Meyer-ter-Vehn T. Klink T. Grehn F. (2016). Conjunctival fibrosis following filtering glaucoma surgery. Exp. Eye Res. 142, 76–82. 10.1016/j.exer.2015.03.021 26675404

[B46] Schmitt E. Paquet C. Beauchemin M. Dever-Bertrand J. Bertrand R. (2000). Characterization of Bax-sigma, a cell death-inducing isoform of Bax. Biochem. Biophys. Res. Commun. 270 (3), 868–879. 10.1006/bbrc.2000.2537 10772918

[B47] Shi Y. Xu X. Zhang Q. Fu G. Mo Z. Wang G. S. (2014). tRNA synthetase counteracts c-Myc to develop functional vasculature. Elife 3, e02349. 10.7554/eLife.02349 24940000 PMC4057782

[B48] Shu D. Y. Lovicu F. J. (2017). Myofibroblast transdifferentiation: The dark force in ocular wound healing and fibrosis. Prog. Retin. Eye Res. 60, 44–65. 10.1016/j.preteyeres.2017.08.001 28807717 PMC5600870

[B49] Suzuki T. Hirokawa T. Maeda A. Harata S. Watanabe K. Yanagita T. (2022). ATR inhibitor AZD6738 increases the sensitivity of colorectal cancer cells to 5-fluorouracil by inhibiting repair of DNA damage. Oncol. Rep. 47 (4), 78. 10.3892/or.2022.8289 35191521 PMC8892626

[B50] Tan J. Liu G. Zhu X. Wu Z. Wang N. Zhou L. (2019). Lentiviral vector-mediated expression of exoenzyme C3 transferase lowers intraocular pressure in monkeys. Mol. Ther. 27 (7), 1327–1338. 10.1016/j.ymthe.2019.04.021 31129118 PMC6612778

[B51] Tan J. Wang X. Cai S. He F. Zhang D. Li D. (2020). C3 transferase-expressing scAAV2 transduces ocular anterior segment tissues and lowers intraocular pressure in mouse and monkey. Mol. Ther. Methods Clin. Dev. 17, 143–155. 10.1016/j.omtm.2019.11.017 31909087 PMC6938898

[B52] Tschesche H. Knäuper V. Krämer S. Michaelis J. Oberhoff R. Reinke H. (1992). Latent collagenase and gelatinase from human neutrophils and their activation. Matrix. Suppl. 1, 245–255. 1480034

[B53] Vaamonde-Garcia C. Malaise O. Charlier E. Deroyer C. Neuville S. Gillet P. (2019). 15-Deoxy-Δ-12, 14-prostaglandin J2 acts cooperatively with prednisolone to reduce TGF-β-induced pro-fibrotic pathways in human osteoarthritis fibroblasts. Biochem. Pharmacol. 165, 66–78. 10.1016/j.bcp.2019.03.039 30936016

[B54] Vendetti F. P. Lau A. Schamus S. Conrads T. P. O'Connor M. J. Bakkenist C. J. (2015). The orally active and bioavailable ATR kinase inhibitor AZD6738 potentiates the anti-tumor effects of cisplatin to resolve ATM-deficient non-small cell lung cancer *in vivo* . Oncotarget 6 (42), 44289–44305. 10.18632/oncotarget.6247 26517239 PMC4792557

[B55] Wang X. Zha M. Zhao X. Jiang P. Du W. Tam A. Y. H. (2013). Siva1 inhibits p53 function by acting as an ARF E3 ubiquitin ligase. Nat. Commun. 4, 1551. 10.1038/ncomms2533 23462994

[B56] Weinreb R. N. Aung T. Medeiros F. A. (2014). The pathophysiology and treatment of glaucoma: A review. JAMA 311 (18), 1901–1911. 10.1001/jama.2014.3192 24825645 PMC4523637

[B57] Willard K. Laguette M. N. Alves de S. R. L. D'Alton C. Nel M. Prince S. (2020). Altered expression of proteoglycan, collagen and growth factor genes in a TGF-β1 stimulated genetic risk model for musculoskeletal soft tissue injuries. J. Sci. Med. Sport 23 (8), 695–700. 10.1016/j.jsams.2020.02.007 32061523

[B58] Wu W. Bonnet S. Shimauchi T. Toro V. Grobs Y. Romanet C. (2022). Potential for inhibition of checkpoint kinases 1/2 in pulmonary fibrosis and secondary pulmonary hypertension. Thorax 77 (3), 247–258. 10.1136/thoraxjnl-2021-217377 34226205

[B59] Xu Y. Jiang Y. Wang Y. Zhao Z. Li T. (2021). LINC00473 rescues human bone marrow mesenchymal stem cells from apoptosis induced by dexamethasone through the PEBP1-mediated Akt/Bad/Bcl-2 signaling pathway. Int. J. Mol. Med. 47 (1), 171–182. 10.3892/ijmm.2020.4788 33236136 PMC7723501

[B60] Yang L. Ho N. Y. Müller F. Strähle U. (2010). Methyl mercury suppresses the formation of the tail primordium in developing zebrafish embryos. Toxicol. Sci. 115 (2), 379–390. 10.1093/toxsci/kfq053 20181659

[B61] Yap T. A. O'Carrigan B. Penney M. S. Lim J. S. Brown J. S. de Miguel L. M. J. (2020). Phase I trial of first-in-class ATR inhibitor M6620 (VX-970) as monotherapy or in combination with carboplatin in patients with advanced solid tumors. J. Clin. Oncol. 38 (27), 3195–3204. 10.1200/JCO.19.02404 32568634 PMC7499606

[B62] Zada M. Pattamatta U. White A. (2018). Modulation of fibroblasts in conjunctival wound healing. Ophthalmology 125 (2), 179–192. 10.1016/j.ophtha.2017.08.028 29079272

[B63] Zhang W. Zhang R. Ge Y. Wang D. Hu Y. Qin X. (2022). S100a16 deficiency prevents hepatic stellate cells activation and liver fibrosis via inhibiting CXCR4 expression. Metabolism. 135, 155271. 10.1016/j.metabol.2022.155271 35914619

[B64] Zhao H. Watkins J. L. Piwnica-Worms H. (2002). Disruption of the checkpoint kinase 1/cell division cycle 25A pathway abrogates ionizing radiation-induced S and G2 checkpoints. Proc. Natl. Acad. Sci. U. S. A. 99 (23), 14795–14800. 10.1073/pnas.182557299 12399544 PMC137498

[B65] Zhou X. Zhang B. Zhao X. Lin Y. Zhuang Y. Guo J. (2022). Chlorogenic acid prevents hyperuricemia nephropathy via regulating TMAO-related gut microbes and inhibiting the PI3K/AKT/mTOR pathway. J. Agric. Food Chem. 70 (33), 10182–10193. 10.1021/acs.jafc.2c03099 35950815

